# Sustainable Bioactive Packaging Based on Thermoplastic Starch and Microalgae

**DOI:** 10.3390/ijms23010178

**Published:** 2021-12-24

**Authors:** Anna Martina Tedeschi, Fabrizio Di Caprio, Antonella Piozzi, Francesca Pagnanelli, Iolanda Francolini

**Affiliations:** Department of Chemistry, Sapienza University of Rome, 00185 Rome, Italy; annamartinatedeschi@gmail.com (A.M.T.); fabrizio.dicaprio@uniroma1.it (F.D.C.); antonella.piozzi@uniroma1.it (A.P.); francesca.pagnanelli@uniroma1.it (F.P.)

**Keywords:** starch, flexible packaging, microalgae, antioxidant packaging, food

## Abstract

This study combines the use of corn starch and *Tetradesmus obliquus* microalgae for the production of antioxidant starch films as flexible packaging material. Starch was plasticized with glycerol and blended with 1 w% polyallylamine chosen as an agent to modify the film physical properties. The addition of polyallylamine improved film water stability and water vapor transmission rate as well as mechanical stiffness and tenacity. The dried *Tetradesmus obliquus* microalgae, which showed an EC_50_ value of 2.8 mg/mg DPPH (2.2-Diphenyl-1-picrylhydrazyl radical), was then used as antioxidant filler. The addition of microalgae provided the films with good antioxidant activity, which increased with microalgae content increasing. To our knowledge, this is the first study reporting the development of sustainable bioactive packaging films composed of almost 100% starch, and follows the European union’s goals on plastics strategy concerning the promotion of bio-based, compostable plastics and the setting up of approaches to prevent food waste with a simple plastic packaging.

## 1. Introduction

Plastic plays a key role in different sectors of the modern society, including in the food sector where plastic packaging helps food preservation, reduces transport cost and overall contributes to lower the environmental impact of food production [1]. Besides these advantages, the high production volume, short usage time and the non-biodegradable nature of most of current plastic packaging arise big concerns about the accumulation of plastic in natural environment (oceans, landfield, etc.) [2]. To move towards a more sustainable economy, the European Union launched a series of actions to tackle plastic pollution and accelerate the transition from a traditional linear economy to a circular economy (https://ec.europa.eu/environment/topics/plastics_en (accessed on 5 December 2021)). Specific EU policies include packaging recycling in a cost-effective manner (https://ec.europa.eu/environment/strategy/circular-economy-action-plan_en (accessed on 5 December 2021)) and the use of bio-based, biodegradable and compostable plastics (https://ec.europa.eu/environment/topics/plastics/bio-based-biodegradable-and-compostable-plastics_en (accessed on 5 December 2021)). In the last two decades, the EU action plans boosted a number of research studies focused on the development of biodegradable plastics from renewable sources in response to the increasing demand of eco-friendly packaging materials. Indeed, the packaging industry, which accounts for about 40% of the total world production of plastics, is now facing a major challenge, that is the replacement of traditional oil-based plastics, like polyethylene (PE), polypropylene (PP), polystyrene (PS) or polyethylene terephthalate (PET), with biodegradable plastics, possibly deriving from renewable resources. In order to replace the traditional plastics in either flexible or rigid packaging, biodegradable polymers should possess a series of requisites like: be light, inexpensive, recyclable, tough, strong and easy to process. Additional characteristics are required for food packaging, where polymers should also have proper barrier properties against permeation by water and waterborne microorganisms to protect food from spoilage and prolong product shelf-life. In this context, the European Committee of the Regions expressed a clear demand for “future research on the relation between packaging and food preservation and possible approaches to prevent food waste without the use of complex plastic packaging” [3].

Among natural biodegradable polymers, in the last two decades, starch has received considerable attention as food packaging material due to its renewability and low cost [4].

The main biodegradable polymers under investigation as alternatives to synthetic oil-based plastics are polylactive (PLA), polyhydroxyalkanoates (PHAs) and starch. A notable feature of PHAs is their production from microbial anaerobic digestion process [5,6]. Starch is instead a carbohydrate consisting of amylose (15–35%) and amylopectin (65–85%), where amylose is a linear polymer of D-glucopyranose units with α- (1 → 4) concatenation, while amylopectin is a highly branched polymer consisting of α- (1 → 4)—D- glucopyranose residues bound through α- (1 → 6) bonds at intervals of about 20 units [7]. Starch is present in different kinds of plants, like corn, potatoes and rice, from which is extracted in granular form. In terms of production costs, starch has a production cost of 0.19 USD/kg [8] and is more convenient than PHAs production (6 USD/kg) [9].

Initially, dry granular starch (GS) was investigated as biodegradable filler for commodity plastics, especially low density polyethylene (LDPE) and linear low density polyethylene (LLDPE), either with or without processing aids [10]. Basically, part of the commodity polymer, generally up to 30%, was replaced by starch granules in order to increase the rate of degradation of plastics products. However, the addition of GS in ductile LDPE had a stiffening effect coupled with a strong elongation decrease [11,12]. The use of modified octenyl succinate starch slightly improved the mechanical properties of the GS/LDPE but decreased the degradation rate [11] while the use of ethylene-co-acrylic acid copolymer as a compatibilizer did not significantly affect the elongation at break or tensile strength, but increased the composite tensile modulus [12]. Starch granule size has also been shown to have an effect on starch-filled PE films, small particle having usually a lower negative impact on PE film properties.

More recently, starch has started to be used as thermoplastic starch (TPS) that is obtained by heating water swollen starch granules up to 90–120 °C, so that the structure of H-bonded starch chains is disrupted, and granule crystallinity is progressively reduced. Following this, so called, gelatinization process [13], starch melts and is free to flow. Upon temperature lowering, the retrogradation process takes place, a phase in which the amylose and amylopectin chains crystallize again giving crystal structures normally different from the initial ones [13]. The resulting water plasticized-TPS has usually physical properties unsuitable for packaging applications, showing poor dimensional stability when moist and a brittle behaviour when dry. Furthermore, water plasticized-TPS tends to recrystallize over time with related shrinkage issues [14]. To improve physical properties of TPS films, TPS has been blended with various plasticizers [14,15]. The most investigated plasticizer for starch is doubtless glycerol because of its good compatibility with starch [15]. However, glycerol effect on film tensile strength and modulus is usually very strong already at low concentrations, negatively affecting resistance to breaking of the material, which *per se* is already low [16,17]. In addition, upon increasing the glycerol content in the film, usually also film susceptibility in water increases [18]. The grafting of multifunctional monomers onto the starch backbone and subsequent crosslinking has been shown to improve both starch water stability of and tensile strength [19,20]. However, most of these reactions are not eco-friendly, although the non-toxic agent citric acid has been lately proposed [21,22].

Blending with synthetic polymers can improve the physico-mechanical properties of TPS. Lately, LDPE/TPS blends, compatibilized with either maleic anhydride-grafted-PE or maleic anhydride-grafted-PP, have been proposed as sustainable alternative to pure polyethylene [23,24]. Although very good results were obtained in terms of elongation at break and tensile modulus, TPS was always the minor component of the blends, reaching the 30% by weight in the best case. Therefore, just a minor part of the blend will biodegrade in a meaningful timeframe.

TPS was also blended with different biodegradable polyesters such as polylactic acid [25], polycaprolactone [26], polyhydroxybutyrate [27] and poly(butylene adipate co-terephthalate) [28]. In these blends, a higher TPS content is used, up to 60%, but, in general, starch/polyesters are incompatible and, therefore, need of suitable compatibilizers, usually starch-grafted-polyesters [29], thus increasing the complexity of the system and also the related costs.

**Scheme 1:**
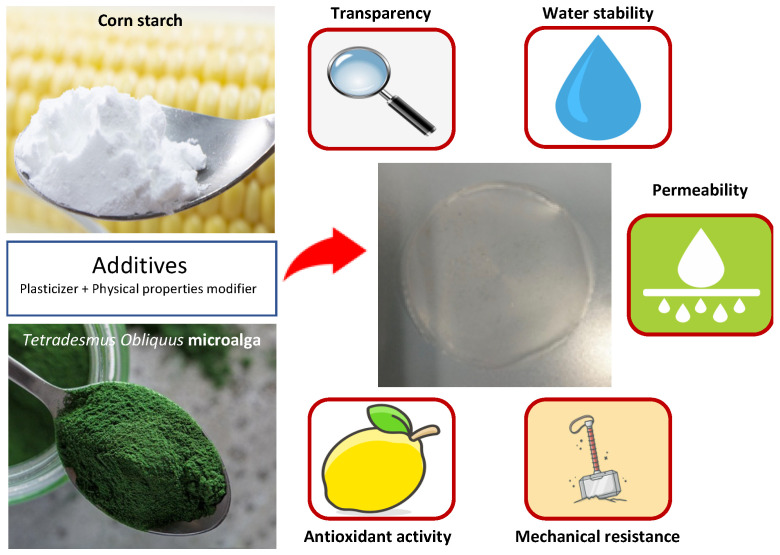
Illustration image highlighting the aims and activities of the study.

The present study aims at producing 100% TPS bioactive films with physico-mechanical properties suitable for packaging applications by combining the biodegradability of starch and the bioactivity of microalgae (Scheme 1). The novelty of the study relies on the setting up of an approach able to modulate the physico-chemical features of 100% TPS films based on the use of a polymeric amine, polyallylamine hydrochloride (PAH), at very low content, as modifier of the film physical properties (Scheme 1). Indeed, it’s known that compounds with aminogroups, like urea [30] and ethanolamine [31], when mixed at low contents (up to 10 w%) with either water or glycerol plasticized-starch, have been shown to have an antiplasticization effect on TPS. The antiplasticization effect is related to the formation of strong H-bonds between the NH groups and OH groups of starch chains competing with the weaker H-bonds established between the starch and plasticizer OH groups [32]. Ethanolamine was also shown to restrains the re-crystallization observed in traditional glycerol or water plasticized-TPS [33]. Our hypothesis was that the negative effects of glycerol on TPS films, that are increasing water susceptibility and lowering tensile strength, could be mitigated by the introduction of an amine compound able to establish strong H-bonds with starch chains. In order to have a strong antiplasticization effect already at low contents, a polymeric amine was chosen in this study. The effect of 1% wt PAH addition into TPS films plasticized with increasing contents of glycerol (Gly, 10, 20 and 30% *w*/*w*) was investigated by evaluating film transparency, water vapor transmission rate, water uptake, soluble fraction, contact angle and mechanical tensile tests.

An additional novelty of the work is the use of dried *Tetradesmus obliquus* microalgae (also known as *Scenedesmus obliquus)* as film bioactive filler. Indeed, *Tetradesmus obliquus* microalgae is known to possesses chlorophylls, carotenoids (lutein, β-carotene) [34] and phenols as antioxidant components [35]. *T. obliquus* was chosen for this study because it is a renewable source that can be easily produced at larger industrial scale to replace fossil derived chemicals with bio-based compounds. As compared to conventional plant sources, *T. obliquus* has the advantage to be cultivable on non-arable lands and with wastewater attaining biomass productivity up to 50–90 t/ha per year [36], almost ten-folds larger than conventional agricultural crops (e.g., soybean). Yet, a previous study indicated *T. obliquus* as the best one in antioxidant properties when compared to other 40 microalgal strains [37]. Although *T. obliquus* has such a relevant potential, its integration in plastic films was studied only in a single previous work [38], while to date the possibility to exploit its bioactive property in a film was not investigated. In this study, the antioxidant activity of the TPS films containing the microalgae was tested versus the 2,2-Diphenyl-1-picrylhydrazyl (DPPH) radical model, along with the effect of the microalgae on the physico-mechanical properties of the TPS films.

## 2. Experimental

### 2.1. Materials

Corn Starch (S, *M*_W_(C_6_H_10_O_5_)_n_ = 218 g/mol, 73% amilopectin and 27% amylose) (Sigma Aldrich s.r.l., Milan, Italy), glycerol (Gly, *M*_W_ = 152.15 g/mol), poly(allylamine) hydrochloride (PAH, *M*_W_ = 50.000 g/mol) (Sigma Aldrich s.r.l., Milan, Italy), 2,2-Diphenyl-1-picrylhydrazyl (DPPH, Sigma Aldrich s.r.l., Milan, Italy), methanol, Potassium nitrate (KNO_3_, Sigma Aldrich s.r.l., Milan, Italy), Sodium sulfate (Na_2_SO_4_, Sigma Aldrich s.r.l., Milan, Italy), Magnesium sulfate (MgSO_4_·7H_2_O, Sigma Aldrich s.r.l., Milan, Italy), Calcium chloride (CaCl_2_·2H_2_O, Sigma Aldrich s.r.l., Milan, Italy), Potassium phosphate dibasic (K_2_HPO_4,_ Sigma Aldrich s.r.l., Milan, Italy), Sodium bicarbonate (NaHCO_3,_ Sigma Aldrich s.r.l., Milan, Italy), Ferric sodium ethylenediaminetetraacetate (NaFeEDTA, Sigma Aldrich s.r.l., Milan, Italy), Ethylenediaminetetraacetic acid disodium salt dihydrate (Na_2_EDTA, Sigma Aldrich s.r.l., Milan, Italy), Magnesium Chloride (MnCl_2_·4H_2_O, Sigma Aldrich s.r.l., Milan, Italy), Zinc sulfate (ZnSO_4_·7H_2_O, Sigma Aldrich s.r.l., Milan, Italy), Cobalt chloride (CoCl_2_·6H_2_O, Sigma Aldrich s.r.l., Milan, Italy), Copper sulfate (CuSO_4_·5H_2_O, Sigma Aldrich s.r.l., Milan, Italy), Sodium molybdate (Na_2_MoO_4_·2H_2_O, Sigma Aldrich s.r.l., Milan, Italy) and 3-methyl-2-benzothiazolinone hydrazone (MBTH) were used as received.

### 2.2. Evaluation of Size and Morphology of Corn Starch Granules

Size and morphology of corn starch granules were evaluated by optical microscopy in polarized light. The optical microscope (OPTIPHOT2-POL, Nikon, Minato, Tokyo, Japan) used in the experiments is equipped with a Linkam HSF 91 thermoregulation device, which permitted to study starch gelatinization in the 25–90 °C temperature range.

### 2.3. Microalgal Biomass Production and Biochemical Characterization

A strain of the microalgae *Tetradesmus obliquus*, generally known as *Scenedesmus obliquus* and recently reclassified by Wynne and Hallan [39], isolated and identified as described in a previously [40] was cultivated in 500 mL column glass photobioreactors (h = 35 cm, d = 5 cm), at 28 ± 1 °C, pH 7.5 ± 0.5, in the following culture medium: KNO_3_ 10 mM; Na_2_SO_4_ 0.7 mM; MgSO_4_·7H_2_O 1 mM; CaCl_2_·2H_2_O 0.5 mM; K_2_HPO_4_ 2.5 mM; NaHCO_3_ 10 mM; NaFeEDTA 28 μM; Na_2_EDTA·2H_2_O 80 μM; MnCl_2_·4H_2_O 19 μM; ZnSO_4_·7H_2_O 4 μM; CoCl_2_·6H_2_O 1.2 μM; CuSO_4_·5H_2_O 1.3 μM; Na_2_MoO_4_·2H_2_O 0.1 μM. The reactors were illuminated 24 h with 35 W led light lamps (equally composed by 6500 K and 4000 K strips) supplying 200 µmol s^−1^ m^−2^ photons and fed with filtered (0.2 µm) 1 L/min air and 10 mL/min CO_2_. The cultivation was repeated in six batches. Initial biomass concentration was set at 0.3 g/L and biomass was harvested (5 min centrifugation at 3000× *g*) after 3 days when 2.4 ± 0.3 g/L biomass was attained. Harvested biomass was rinsed twice with distilled water and dried at 50 °C overnight. The resulting powder was reduced in size by grinding in an agata mortar and sieved through a 90 µm sieve. Biochemical characterizations were performed on the dry powder as follows. Proteins were determined as N% 6.25, based on N% determined with an elemental analyzer (EA 1110 CHNS/O). Starch was determined by using the total starch assay kit (Megazyme, NEOGEN Europe Ltd., The Dairy School Auchincruive, Ayr, KA6 5HU, Scotland, United Kingdom), following the protocol as described by de Jaeger et al. [41]. Total carbohydrates were determined by acid hydrolysis followed by spectrophotometric analysis of sugars by using the 3-methyl-2-benzothiazolinone hydrazone (MBTH), following the official method published by the NREL (NREL/TP-5100-60957) [42]. Lipids were determined by the Folch method [43], modified by using 2:1 dichloromethane:methanol as organic solvent. After extraction, total lipids were quantified gravimetrically after solvent evaporation under N_2_ flux. Pigments (chlorophylls + carotenoids) were extracted from 5 mg biomass with methanol at 70 °C for 10 min and quantified with the equation reported by Lichtenthaler [44]. After measuring the absorbance at 665.2 nm, 652.4 nm and 470 nm by an UV-Vis spectrophotometer (Cary 50 Scan, Varian Australia Ltd., 679 Springvale Road Mulgrave, VIC 3170 Australia).

### 2.4. Preparation of Bioactive Starch-Based 2D-Matrices

Starch 2D-matrices (films) were prepared by the solvent casting and evaporation method. First, 2% *w*/*v* starch suspension was submitted to a process of gelatinization at 90° for 30 min under magnetic stirring (500 rpm) and then to retrogradation by lowering temperature up to 40 °C. At this stage, additives were incorporated in the starch solution. Following 20 min of stirring at 40 °C, the solution (25 mL) was poured into a Teflon Petri dish (6 cm in diameter) and dried for 48 hr at room temperature to obtain thin films.

Three sets of films were obtained according to the types of additive mixed with starch.

Set 1: Starch films (S) containing glycerol (Gly) as plasticizer. Films were named S_GlyX where X stands for the amount of glycerol (10, 20 and 30% *w*/*w*).

Set 2: Starch films containing glycerol plus poly(allylamine) hydrochloride (PAH, 1%) as film property modifier (antiplasticization agent). Films were named S_PAH1_GlyX where X stands for the amount of glycerol (10, 20 and 30% *w*/*w*).

Set 3: Starch films containing glycerol (30%), poly(allylamine) hydrochloride (1%) and dried microalgae *Tetradesmus obliquus* as bioactive filler. Films were named S_PAH1_Gly30_Y where 30 is the glycerol content and Y stands for microalgae content (0.1, 0.5 and 1% *w*/*w*).

The whole microalgal biomass was used as filler in order to exploit the full bioactive property of microalgae biomolecules. Instead, the utilization of pure extracts would make loss some other biomolecules, synergic effects among biomolecules and it could increase their degradation rate.

### 2.5. Film Thickness

Thickness of the films was determined by a digital caliber, making three measurements on three distinct points for each film.

### 2.6. Fourier-Transform Infrared Spectroscopy

Fourier-transform Infrared Spectroscopy (FTIR) was performed in Attenuated Total Reflection (ATR) by a Nicolet 6700 (Thermo Fisher Scientific, Waltham, MA, USA) equipped with a Golden Gate ATR accessory (angle of incidence 45°), at a resolution of 4 cm^−1^ and co-adding 200 scans.

### 2.7. Thermogravimetric Analysis (TGA)

Thermal stability of starch films was evaluated by thermogravimetric analysis (TGA) by employing a Mettler TG 50 thermobalance. A thermal scanning interval of 25–500 °C and a scanning speed of 10 °C/min were set. Analyses were carried out under nitrogen flow, on 5–8 mg sample weight. The degradation temperature was determined in correspondence of the peak of the first derivative of the weight with respect to temperature.

### 2.8. Film Transparency

The ultraviolet and visible light barrier properties of the film were analyzed by a spectrophotometer HP DIODE ARRAY (HP8452A, Hewlett Packard, Palo Alto, CA, USA), following the ASTM D1746-09 standard procedure (Standard Test Method for Transparency of Plastic Sheeting). Measurements were carried out in absorbance mode at 2 nm intervals in the 190–820 nm range. The transparency of the films was calculated from the percent transmittance (T%) of light at 580 nm, according to the following equation:

$$T\;\left(\%\right)=\frac{10^{\left(2\;-\;A\right)}}{x}$$

where A is the absorbance at 580 nm and x is the film thickness.

Three parallel experiments were performed for each film and data were reported as value ± standard deviation.

### 2.9. Water Vapor Transmission Rate

Water vapor permeability tests were carried out following the ASTM method E96 (Standard Test Methods for Water Vapor Transmission of Materials) with some modifications. Circular films were sealed as a patch onto a glass container (diameter = 1.4 cm and height = 5 cm) containing 10 mL of water. The weight of the assembled system was monitored over time, under constant temperature, and the water vapor transmission rate (WVTR) was determined according to the equation [45]:

$$\mathrm{WVTR}\;\left(\frac{g}{h\cdot m^{2}\;\cdot \mathrm{mm}}\right)=\frac{W}{t\cdot S\cdot T}$$

where W is the system weight (g), t is time (h), S is the film surface area (m^2^) and T is the film thickness (mm).

Experiments were performed in triplicate.

### 2.10. Water Uptake and Soluble Fraction

The swelling ability of polymer films was evaluated by immersing weighted films in water for increasing times up to 2 h. At determined times, films were collected and weighted after removal of excess solvent by a filter paper. Water uptake was evaluated as follows:

$$\mathrm{WA}\;\left(\%\right)=\left(\frac{W_{t}-{\;W}_{0}}{W_{0}}\right)\cdot 100$$

where W_t_ is the sample weight at the time t and W_0_ is the initial sample weight.

For soluble fraction (SF) determination, 2 h-swollen films were dried at room temperature up to constant weight and weighted. SF was determined as:

$$\mathrm{SF}\;\left(\%\right)=\left(\frac{W_{0}-{\;W}_{d}}{W_{0}}\right)\cdot 100$$

where W_d_ is the sample weight after drying and W_0_ is the initial sample weight.

Three parallel experiments were performed.

### 2.11. Static Contact Angle Determination

To determine surface wettability of starch films, the static contact angle was evaluated by the drop method. Particularly, a water droplet (Milli-Q-water) was deposited onto the film surface and a picture was captured. The resulting image was processed by Software Motic Image Plus 2.0 ML in order to determine the contact angle (CA) as follows:

$$\mathrm{CA}\;\left(\theta \right)={\;\tan}^{-1}\;\frac{2\;h}{D}$$

where D is the drop base length and h is the drop height.

### 2.12. Mechanical Tests

Mechanical features like Tensile Strength (TS), Young’s Modulus (E), Elongation at break (ε) and Tenacity (T) of films were determined by tensile tests by using an ISTRON 4502 instrument (Instron Inc., Norwood, MA, USA). For the analysis, films were cut into rectangular specimens (6 cm × 1 cm × 0.1 cm length × width × thickness) and fixed between the two Instron flat plates. Measurements were carried out at 100 mm/min deformation rate and with a 2 kN load cell.

### 2.13. Antioxidant Properties of Films by DPPH Test

The antioxidant activity of films containing *Tetradesmus obliquus* microalgae as well as of the pure microalgae powder was evaluated by assessing the material ability to scavenge the DPPH (2,2-Diphenyl-1-picrylhydrazyl) free radical [46]. DPPH is a stable radical giving a violet solution, which turns to yellow when DPPH is reduced to DPPH-H by an antioxidant compound. This reaction can be followed by UV-vis spectroscopy by monitoring the decrease in the absorbance at 514 nm.

For each material, different concentrations, expressed as material weight (mg) per DPPH (mol), were assayed. Specifically, a weighted amount of sample, either polymer film or microalgae powder, was put in contact with a DPPH solution in methanol (2 mL, 2 mM). After 30 min, the suspension was centrifuged, the supernatant was collected and submitted to UV-Vis spectroscopy. The amount of residual DPPH was determined by a previously obtained calibration curve (Abs vs. DPPH concentration), while the Scavenging Activity (Q, %) for each sample amount was defined as:

$$Q\;\left(\%\right)=\left(\frac{A_{0}-\;A}{A_{0}}\right)\cdot 100$$

where A_0_ is the absorbance of the initial DPPH solution while A is the absorbance of the solution after reaction with the antioxidant material.

The scavenging activity was then plotted vs. the antioxidant concentration (mg/DPPH mol) to determine the Efficient Concentration (EC_50_) defined as the amount of compound able to decrease by 50% the initial DPPH concentration [47]. A starch film without microalgae was used as control.

### 2.14. Statistics

One-way analysis of variance comparisons was performed using MiniTab. Differences were considered significant for *p* values < 0.05. Data were reported as means ± Standard Deviation.

## 3. Results and Discussions

**Figure 1:**
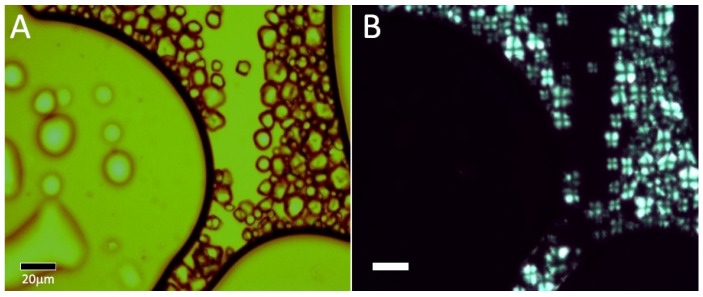
Optical microscopy (OM) images of starch granules under normal light (**A**) and polarized light (**B**).

Starch (S) is a polysaccharide deriving from different kinds of plants, from which is extracted in granular form. The size and morphology of starch granules strongly depends on the botanical source and may range from 2–3 μm to ca. 100 μm [48]. The corn starch granules used in this study shows predominantly a spherical morphology and homogeneous size ranging from 10 to 25 μm (Figure 1), this finding being consistent with literature data [49,50].

Under polarized light (Figure 1B), the Maltese cross birefringence pattern, typical of crystalline materials, was observed. Starch crystallinity results from hydrogen bonds holding starch chains together and is responsible for insolubility of starch in cold water. Starch crystallinity also affects degradability rate of starch and depends on the ratio between contents of amylose, a linear polysaccharide, and amylopectin, a branched polysaccharide [51].

**Figure 2:**
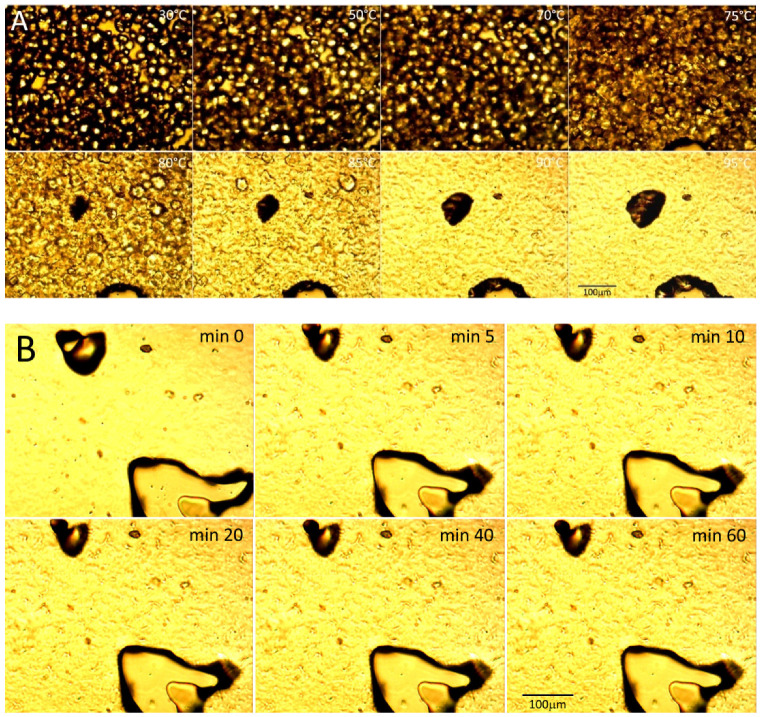
(**A**) Starch gelatinization: OM images of starch granules at temperatures ranging from 30 °C to 95 °C. (**B**) Starch retrogradation: OM images of starch granules at 40 °C over time.

In order to set up the procedure for preparation of starch/additives compounds, the processes of starch gelatinization and retrogradation were followed by optical microscopy (OM) by heating the sample from 30 °C to 95° C at a 20 °C/min speed. In Figure 2, the structural variations of the granules during gelatinization as a function of the temperature (Figure 2A) and during retrogradation (Figure 2B) at 40 °C are shown.

As it can be observed, gelatinization begins at ca. 75 °C and resulted to be complete at ca. 90 °C. By lowering temperature at 40 °C, set as the retrogradation temperature, granules’ recrystallization was not significant for the first 60 min. Therefore, the temperature of 90 °C was adopted for the starch gelatinization phase while 40 °C was chosen as the temperature for mixing the additives to the starch solution.

### 3.1. Preparation of Starch Films and Their Characterization

In order to investigate the effect of polyallylamine chloride (PAH) on the physico-mechanical features of glycerol-plasticized starch films as well as to endow the films with antioxidant properties, in this study, three sets of starch films were prepared: (1) the S_GlyX series, containing only glycerol (Gly) as plasticizer at either 10, 20 or 30% *w*/*w* content (X); (2) the S_PAH1_GlyX series, containing 1% *w*/*w* PAH plus glycerol (10, 20 or 30%); and (3) the S_PAH1_Gly30_Y series, containing 1% *w*/*w* PAH, 30% *w*/*w* Gly and dried microalgae *Tetradesmus obliquus* as bioactive filler at either 0.1, 0.5 or 1% content (Y). The obtained films were physico-chemically characterized in order to find out a correlation between their performance and the additive type and content.

#### 3.1.1. Film Appearance and Optical Properties

Flexible packaging films should be transparent and light in color to meet the aesthetic needs of consumers. However, light can accelerate photochemical degradation reactions that induce food deterioration [52]. In general, film transparency is influenced by several factors, such as the degree of crystallinity of the material and material heterogeneity (presence of fillers). In this study, film transparency was investigated both visually and through determination of the transmittance (T%) of light at 580 nm, normalized for film thicknesses [53].

**Figure 3:**
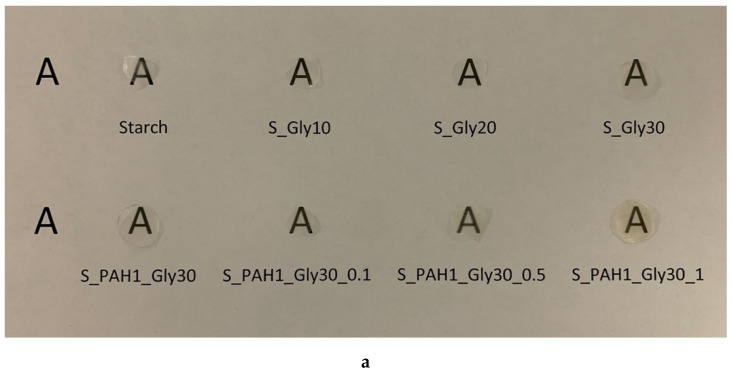

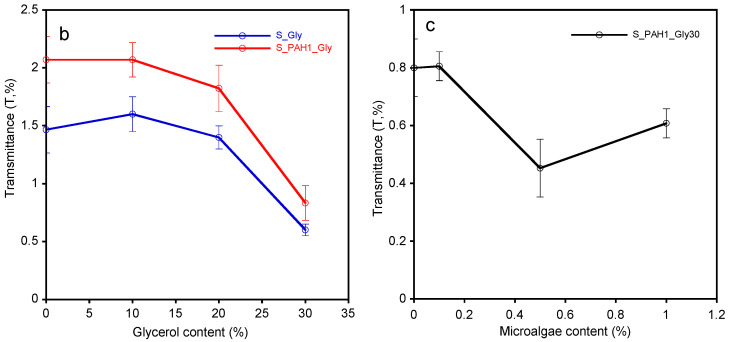
Film transparency properties. Digital images of films obtained with different formulation (**a**); Transmittance of films as a function of glycerol content with or without 1% w PAH (**b**); Transmittance of S_PAH1_Gly30 film as a function of microalgae content (0.1, 0.5 and 1%) (**c**).

In Figure 3a, digital images of films obtained with the different formulation are reported. As it can be seen, in any case, homogenous and quite transparent films were obtained. Film plasticization with glycerol (Gly) seemed to slightly improve film transparency at least for 10% Gly contents (Figure 3a). That was confirmed by UV-vis spectroscopy analysis, which showed an increase in transmittance for the sample S_Gly10 with respect to the plain Starch film (*p* < 0.05, Figure 3b). Such finding could be related to the decrease in starch crystallinity induced by glycerol. Indeed, semi-crystalline polymer films, usually, exhibit a reduced transparency compared to amorphous films due to the different refractive indexes of the amorphous and crystalline phases, which generate light scattering [54]. For higher Gly contents, film transmittance decreased, especially when 30% Gly was introduced in the film (S_Gly30). Presumably, partial segregation of the plasticizer occurred, thus making the material heterogeneous.

The introduction of 1% wt PAH in the films improved film transparency at all Gly contents (Figure 3b). Higher values of T (%) indicate greater homogeneity of films containing PAH, suggesting a good compatibility of PAH with starch. Presumably, the presence of PAH may have disturbed starch crystallization during the retrogradation phase.

As far as the starch films containing the *Tetradesmus obliquus* microalgae, the transparency was studied only for the S_PAH1_Gly30 film, containing 1% PAH and 30% Gly, because this film was shown to possess the best physico-mechanical properties as will be widely described later on. As can be observed in Figure 3c, a decrease in the transmittance was observed, related to sample heterogeneity. Yet, microalgae contain several pigments, that has a certain light absorbance at 580 nm, contributing on reducing the transmittance. The same pigments gave a slightly yellow brownish color to the films (Figure 3a). Although pigments could be potentially removed from microalgae by a solvent extraction pre-treatment, this was not considered useful for the purpose of this study because pigments are among the main microalgal bioactive compounds [55]. Anyway, both the physical appearance and the transparency of the films containing the microalgae remained acceptable for the application.

#### 3.1.2. Study of Starch/Additive (Gly and PAH) Interactions by FTIR

**Figure 4:**
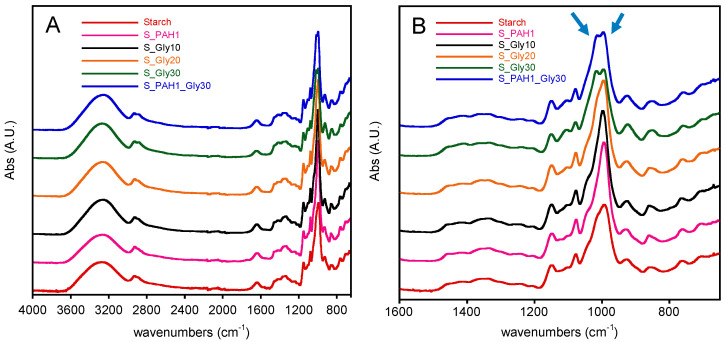
FTIR spectra (**A**) of starch, glycerol-plasticized starch films (S_Gly10, S_Gly20 and S_Gly30), PAH containing-starch film (S_PAH1) and starch film containing both 1% PAH and 30% Gly (S_PAH1_Gly30). Enlargement of the 1600–650 cm-1 spectral range (**B**). Arrows indicate peaks at 995 cm^−1^ and 1015 cm^−1^.

Figure 4 shows the FTIR spectra of pure starch, glycerol-plasticized starch and PAH-containing starch. In the starch FTIR spectrum (Figure 4A), the adsorption band located between 3600 and 3200 cm^−1^ is associated with the free hydroxyl groups, whereas peaks in the range 3000–2800 cm^−1^ are associated to the C-H stretching and the peak at 1639 cm^−1^ is attributed to the bending of the bond (H-O-H) of the absorbed water.

In the spectral region between 1200 and 900 cm^−1^, the bands related to C-O-C, and C-O-H vibrations are present while at 1334 cm^−1^ the bending vibrations of the bond (C-O-H) are observed [56].

Very small differences in the profile shape of peaks can be observed in the starch fingerprint, and particularly in the 1100–900 cm^−1^ region, as a result of starch interactions with glycerol or PAH. Specifically, around 1000 cm^−1^, three bands are present in the starch spectrum at 1042 cm^−1^ (shoulder), 1015 cm^−1^ (shoulder) and at 995 cm^−1^ (peak). When glycerol was added in the films, the peak at 995 cm^−1^, related to C-OH stretching, was shifted to 998 cm^−1^ (Figure 4, blue arrows). In addition, for the sample containing the highest Gly amount (30%), an increase in the intensity of the adsorption at 1015 cm^−1^ was observed. These changes, not observed when only PAH was present (S_PAH1, Figure 4), may be associated to variation in starch crystallinity induced by starch interaction with Gly. Indeed, according to Vicentini et al. [57] and Goodfellow and Wilson [58], polysaccharides adsorption peaks in the 1300–900 cm^−1^ spectral range are more sensitive to molecular conformations than those in the 1400–1300 cm^−1^ region, and variation in the peaks around 1000 cm^−1^ can be associated to crystalline-amorphous transition. Therefore, FTIR analysis confirmed the plasticization effect of glycerol, very strong when Gly was present at 30% wt, but did not provide information about PAH interactions with starch presumably due to the very low PAH content (1%) present in the films.

#### 3.1.3. Thermogravimetric Analysis of S_GlyX and S_PAH1_GlyX Films

**Figure 5:**
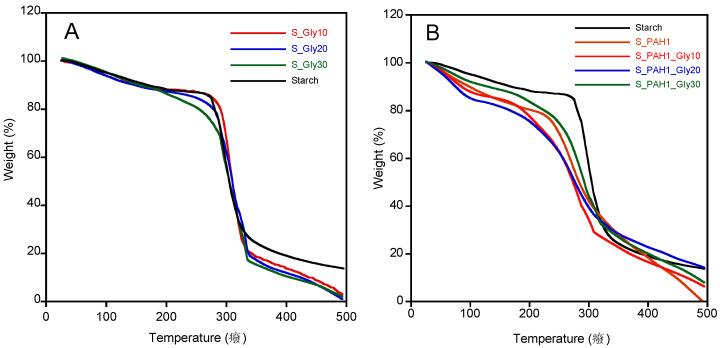
Thermogravimetric curves of S_GlyX (**A**) and S_PAH1-GlyX (**B**) films.

Thermogravimetric analysis (TGA) was carried out to determine the thermal stability of the different starch formulations and thus gaining information about the establishment of potential interactions between starch and additives. Figure 5 shows the thermo-gravimetric curves of pure glycerol-plasticized films (Figure 5A) and containing PAH 1% wt (Figure 5B).

In the thermogram of the native starch film, a first weight loss of 10% is observable at around 100 °C related to evaporation of absorbed water and a second weight loss at ca. 300 °C related to polymer chain degradation. The presence of Gly at all of the explored contents did not affect significantly starch thermal stability, although a weight loss in the 200–250 °C temperature range is present in the S_Gly20 and S_Gly30 samples (Figure 5A), presumably related to degradation of the glycerol not interacting with the starch chains, suggesting a segregation of the plasticizer into small clusters for contents equal to or higher than 20% [59].

The possibility of establishing strong interactions between plasticizers and an auxiliary component has been already explored in the literature for several purposes, like the control of drug release in pharmaceutical formulations [60] or preservation of activity of biomolecules [61]. In these studies, the stability imparted to the systems by the auxiliary component was related to the antiplasticization activity of the auxiliary component itself.

#### 3.1.4. Soluble Fraction, Water Swelling and Contact Angle of S_GlyX and S_PAH1_GlyX Films

The study of film behavior in water environment is very important for packaging application [62,63].

In Table 1, the soluble fractions of pure glycerol-plasticized films and added with 1% wt PAH are reported. In the glycerol-plasticized films (S_GlyX), the soluble fraction increased with plasticizer content increasing. This is an effect known in the literature [64], as polyols essentially weaken the interactions between polymer chains by increasing the free volume and this promotes the diffusion of water between polymer chains and the solubilization of higher portions of biopolymer. It is possible that glycerol interacts preferentially with amylopectin chains, which being branched have a more open structure than linear amylose chains and can therefore better allow the penetration of small molecules. The same considerations have been called into question in the literature to justify the insolubility in water of amylose chains, with respect to amylopectin chains.

As far as films containing PAH are concerned, the non-plasticized sample (S_PAH1) shows a very low soluble fraction, even lower than the pure starch film, related to the antiplasticization effect of PAH consisting in the formation of strong H-bonds between PAH NH-groups and starch OH groups [30]. When glycerol was introduced, as expected, the soluble fraction increased but a correlation between the soluble fraction and plasticizer content couldn’t be found anymore. Indeed, the soluble fraction was found to be ca. 20% for all of the S_PAH1_GlyX films. Interestingly, by comparing the films with 30% Gly content with or without PAH, a strong stabilization effect by PAH can be observed, the soluble fraction decreasing from 35% of S_Gly30 to 22% of S_PAH1_Gly30. This finding agrees with thermal analysis, where the S_PAH1_Gly30 film was found to be the most thermally stable, and further supports the hypothesis of the formation of strong glycerol-PAH-starch interactions at the 30% Gly content.

Water swelling measurements and contact angle determination also evidenced a peculiar behavior of the S_PAH1_Gly30 film. Indeed, this film showed the lowest equilibrium swelling percentage (90%, Table 1) and the highest contact angle value (84°, Table 1), confirming the less propensity of this film to interact with water with respect to the other formulations. In absence of PAH, the addition of Gly in starch films increased slightly the equilibrium swelling degree (from 110% of pure starch to 125% of S_Gly30) and significantly lowered the contact angle (from 85° of pure starch to 61° of S_Gly30). These effects are always related to the plasticization activity of Gly.

#### 3.1.5. Evaluation of Water Vapor Transmission Rate (WVTR)

Water vapor permeability is an important feature for food packaging application. Usually, low permeability values are required for packaging films in order to reduce the transfer of moisture from the environment to food and to ensure longer storage times [65]. However, in case of some types of fresh foods, plastics with high permeability to oxygen and water vapor are required for the packaging to avoid condensation of water vapor inside the packaging, which may lead to microbial growth and food spoilage [66].

Gas transmission through a packaging material can take place mainly by two mechanisms: (i) pore effect, for which the gas crosses the material through small holes or cracks of the structure; (ii) solubility-diffusion effect, for which gas transmission is related to the concentration gradient between the two packaging sides and the gas solubility in the material. Gas diffusion depends on the characteristics of both the penetrating molecule (size, polarity) and the packaging material (crystallinity, degree of cross-linking and polymer chain segmental motion) [67]. The presence of additive in the main packaging material can affect material permeability either by changing gas solubility in the matrix or by creation of nanoholes in the structure [68]. The effects of additives on material permeability, however, cannot be generalized and depend on the properties of the used additive/material couple.

Table 1 reports the WVTR values of S_GlyX and S_PAH1_GlyX films. The S_GlyX film series shows a decrease in WVTR with the glycerol content increasing. This is an unexpected result, although some authors have shown a similar decrease in WVTR for Gly content up to 20% or 30%, while an increase in WVTR was found for greater Gly contents [18,69]. Presumably, the strong affinity of the Gly plasticized-starch matrices for water (see water swelling and contact angle, Table 1) limits water diffusion, through the matrix itself, and release on the other side of the film. When also PAH was present in the films (S_PAH1_GlyX), WVTR values higher than those of films containing only Gly (S_GlyX) were observed, at all Gly contents (Table 1). That finding may be related both to the higher hydrophobicity of the S_PAH1_GlyX samples (see contact angle, Table 1), which may decrease the matrix’s ability to retain water, as well as to the PAH ability to disturb inter-chain starch/starch interactions, already hypothesized in the discussion of TGA data, which may favor the diffusion of water in the matrix.

In general, our films showed WVTR values higher than LDPE, that is the most used polymer in flexible packaging. Indeed, LDPE WVTR values range from 6.7 and 8.7 g/m^2^ day [68] while in our case WVTR is in the range 12–77 g/m^2^ day for S_GlyX films and 55–77 g/m^2^ day for S_PAH1_GlyX films. Therefore, the use of S_PAH1_GlyX films should be limited for the packaging of fresh foods characterized by high transpiration rate such as strawberries, asparagus, mushrooms [70], for which a 33–68 g/m^2^ day target water vapor transmission rate of packaging materials has been suggested to avoid condensation of water vapor inside the packaging [71]. Indeed, without a suitable packaging, fruit release large amounts of water vapor that, if build-up inside the package, facilitates microbial growth [72].

#### 3.1.6. Mechanical Properties of S_GlyX and S_PAH1_GlyX Films

**Figure 6:**
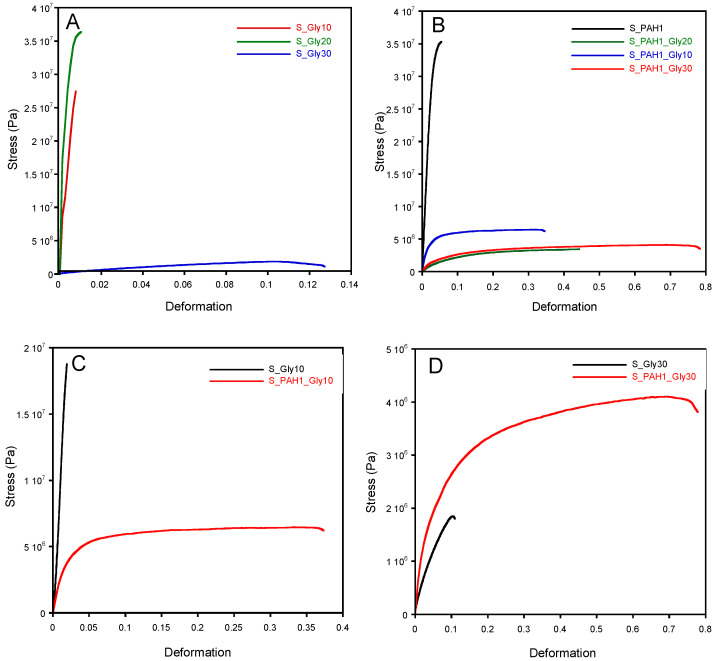
Stress-strain curves of S_GlyX films (**A**), S_PAH1_GlyX films (**B**) and comparison of stress strain curves with or without PAH at 10% (**C**) and 30% (**D**) Gly content.

The effects of glycerol and PAH on the mechanical properties of the films were investigated by performing tensile tests. In Figure 6, the stress-strain curves of the sample are shown while in Table 2 the Young Modulus (E), the strength at break, elongation at break and tenacity of films are reported. It was not possible to perform tensile tests on the pure starch film because of its excessive brittleness.

As for as the S_Gly series is concerned (Figure 6A), a slight increase in the elongation at break was observed with the increase in the plasticizer content, from 3% of S_Gly10 to 13% of S_Gly30 (Table 2). However, at the highest Gly content (30%), the film Young modulus, Strength at break and Tenacity decreased strongly (Table 2), that being the negative side of using glycerol [16,17]. When PAH1 was introduced in the films together with Gly (S_PAH1_GlyX), an improvement in the elongation at break was observed both at low (10%, Figure 6C) and at high (30%, Figure 6D) Gly content, confirming that PAH interferes with the inter-chain starch-starch interactions.

Interestingly, the S_PAH1_Gly30 sample not only had a ductility higher than S_Gly30, but also showed greater Young modulus, strength at break and tenacity (Table 2), highlighting the ability of PAH to stabilize the film structure presumably throughout the formation of strong H-bonds between its NH groups and OH groups of both starch and glycerol.

The S_PAH1_Gly30 film has the tensile strength (4 MPa), elastic modulus (60 MPa) and elongation at break (ca. 80%) lower than LDPE (TS = 7–25 MPa, E = 150–340 MPa, EB = 300–900%) [68] but of the same order of magnitude. Therefore, under a mechanical point of view, the S_PAH1_Gly30 film seems to be a good candidate for replacement of LDPE in flexible packaging. Due to its good physical properties, the S_PAH1_Gly30 film was chosen for the further experiments involving the entrapping of *T. obliquus* microalgae as antioxidant filler.

**Scheme 2:**
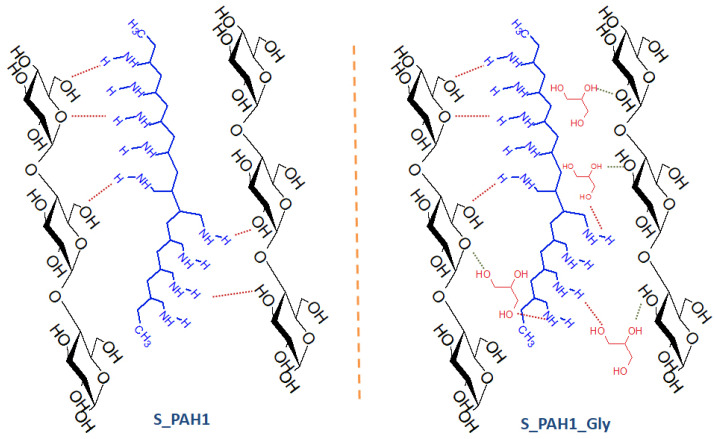
Hypothesis of structure of the starch-PAH composite (S_PAH1) and starch-PAH-Glycerol composite (S_PAH1_Gly). The green dotted lines represent weak H-bonding while the red dotted lines weak represent strong H-bonding.

In the Scheme 2, a hypothesis of the structure of the composites under study is reported.

Both the plasticizer glycerol and the anti-plasticizer PAH can establish hydrogen bonds with the starch in replacement of the water-starch H-bonding and starch-starch inter- and intra-molecular H-bond interactions. However, the strength of the PAH-starch H-bonding was hypothesized to be higher than that established between starch and glycerol, based on the electronegativity of the pair of acceptor and donor involved in H-bonding. Indeed, if starch is the acceptor of the hydrogen, with either C-O-C glycosidic linkage or a hydroxyl group, the hydrogen-donating effect of the amine group in PAH is higher than that of glycerol or water. On the contrary, if starch is the H-donor, with is OH groups, the electronegativity of the PAH nitrogen is lower than that of oxygen in glycerol. Therefore, in the competition of H-bonding with starch, glycerol is always weaker than PAH, thus justifying the higher tenacity of the S_PAH_Gly films with respect to the S_Gly ones (Table 2). 

### 3.2. Characterization of S_PAH1_Gly30 Films Containing T. obliquus Microalgae

#### 3.2.1. Physico-Mechanical Properties

Microalgae are photosynthetic microorganisms often exposed to conditions inducing oxidant stress during growth, as high O_2_ and light levels, which could induce photoinhibition. *T. obliquus* is a fast-growing species able to grow under high light intensity with low sensitivity to photoinhibition [73]. To face photoinhibition, algae have developed protection systems based on carotenoids, phenols and other antioxidant biomolecules to prevent the accumulation of free radicals and species reactive to oxygen. *T. obliquus* contains different bioactive molecules such as pigments (carotenoids and chlorophylls), phenols, proteins, polysaccharides and fatty acids [74]. These biomolecules give to *T. obliquus* antioxidant and antiviral properties [75]. For these reasons, *T. obliquus* was used in this study as antioxidant filler for the S_PAH1_Gly30 film. *T. obliquus* was produced from cultivation under optimal environmental conditions, for inducing high growth rate, high photosynthetic yield and high content of pigments and phenols [73]. Biomass harvesting was done in correspondence of the point at which nitrogen was depleted from the culture medium, which corresponds with the maximum productivity for antioxidants as phenols and pigments [35,76]. The biochemical composition shows a predominant content of proteins (54 ± 3%), typical for cells grown up in nutrient replete conditions. The sum of the three macro-components total carbohydrates (16 ± 3%, of which starch accounts for 8 ± 1%), lipids (20 ± 2%) and proteins (54 ± 3%) gave 90 ± 8%. The residual fraction was composed by ash and sporopollenin (also known as algaenan), which were not analyzed, but are typically around 1–10%. The difference between the percent content of starch and total carbohydrates is mainly due to cell wall polysaccharides made of hemicelluloses and cellulose. Phenols were not measured in this study, but it is known that this strain in these conditions contains about 10 mg/g phenolic compounds [35].

Expect starch (8%) and ash (1–3%), all the other microalgal components are well known to have antioxidant and other bioactive properties (e.g., antiviral) [74,77]. In order to exploit its full antioxidant potential, the whole biomass was used as a filler in this study. This approach also allows to exploit synergic interactions among different biomolecules and to avoid any further biomass processing, that may be unfeasible when the production process is scaled up at industrial level.

**Figure 7:**
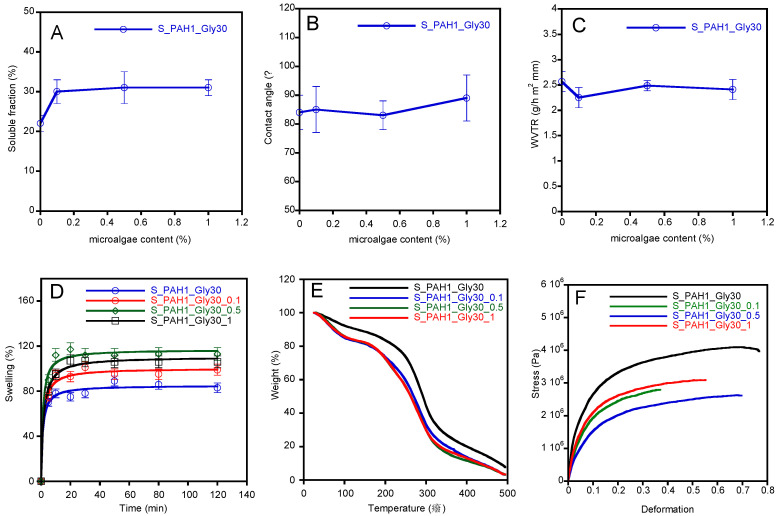
Physicochemical properties of S_PAH1_Gly30 films containing different microalgae amounts (0.1, 0.5 and 1% wt). Soluble fraction (**A**), Contact angle (**B**), water vapour transmission rate (**C**), swelling in water (**D**), thermogravimetric curve (**E**) and Stress-strain curve (**F**).

In Figure 7, the physicochemical properties of S_PAH1_Gly30 film containing different amounts (0.1, 0.5 and 1% wt) of *T. obliquus* microalgae are reported. As it can be seen, the introduction of microalgae did not significantly affect the film soluble fraction (Figure 7A), wettability (Figure 7B), and WVTR (Figure 7C). In contrast, a slight increase in the water swelling degree (Figure 7D) as well as a decrease in thermal stability (Figure 7E) and mechanical resistance (Figure 7F) were observed and related to sample heterogeneity. However, the mechanical resistance of the S_PAH1_Gly30 films containing microalgae remained comparable to that of the pure film. As an example, the S_PAH1_Gly30_1 film, with the highest microalgae content, possessed a Young modulus of 30 MPa (vs. 63 MPa of S_PAH1_Gly30), a strength at break of 3.1 MPa (vs. 4 MPa of S_PAH1_Gly30) and an elongation at break of 55% (vs. 78% of S_PAH1_Gly30).

#### 3.2.2. Antioxidant Activity

*Tetradesmus obliquus* microalgae have shown an antioxidant power greater than other microalgae both eukaryotic and prokaryotic [37]. The high antioxidant activity of *T. obliquus* microalgae was confirmed in this study by using DPPH as radical model. Specifically, the microalgae possessed an EC_50_ value of 1100 mg/mmol DPPH (2.8 mg/mg DPPH) corresponding to 2.2 mg/mL. Unfortunately, it’s difficult to compare the obtained EC_50_ value with literature data. Indeed, the antioxidant activity of *T. obliquus* (*Scenedesmus obliquus*), reported by Guedes and colleagues [37], ranged from 2 to 63 mg/L, in dependence on the investigated microalgae species, but was expressed in ascorbic acid equivalent. In addition, the ABTS radical was used instead of DPPH.

**Figure 8:**
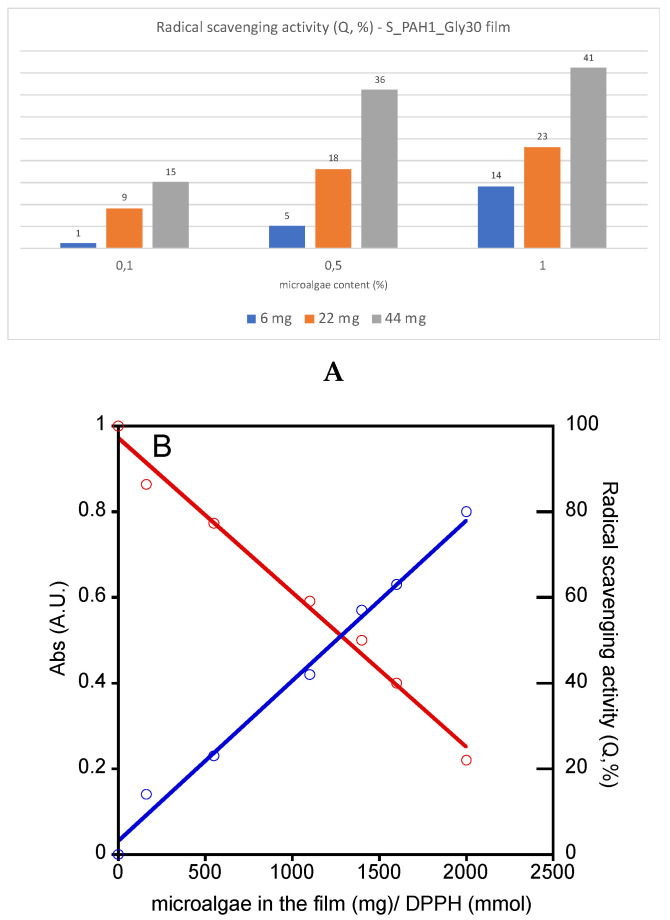
Radical scavenging activity (Q) of *T. obliquus* microalgae entrapped in the S_PAH1_Gly30 film as a function of microalgae content (**A**); Double Y plot for the determination of the EC_50_ value of the S_PAH1_Gly30_1 film (**B**).

Once entrapped in the S_PAH1_Gly30 film, the microalga was able to maintain its activity, as shown in Figure 8A where the radical scavenging activity (Q, %) of the S_PAH1_Gly30 films containing the microalgae is reported. As expected, for equal film amount (6.5 mg, 22 mg or 44 mg), the antioxidant activity of the film increased with the microalgae content increasing. This result agrees with a previous study reporting that the amount of *Heterochlorella luteoviridis* and *Dunaliella tertiolecta* biomass in the starch film reduced proportionally the oxidation rate of oil during storage [78]. The starch without the microalgae showed very low radical scavenging activity (less than 10%) in the range of investigated material amounts.

For the most active sample S_PAH1_Gly30_1, the EC_50_ value of the microalgae entrapped in the film was determined by plotting the film radical scavenging activity vs. the amount (mg) of microalgae present in the film normalized for DPPH amount (mmol) (Figure 8B).

Interestingly, when entrapped in the starch film, the microalgae showed an EC_50_ value of 1252 mg/mmol DPPH very similar to the EC_50_ value it showed when tested as a free powder. This finding suggests a good interaction of the DPPH radical with the microalgae when entrapped in the starch film, which is presumably related to a good dispersion of the microalgae in the film itself. These good results are promising in the view of the exploitation of *T. obliquus* also for other bioactive properties, as the antiviral activity, which is worth of investigation in future studies.

## 4. Conclusions

In this study, an approach to obtain bioactive 100% TPS flexible films with physico-mechanical properties as much as closer to LDPE has been set up. The use of a polymeric amine, polyallylamine, in a very low content (1%) in glycerol-plasticized starch films permitted to improve film water stability, transparency and water vapor transpirability as well as to increase the film mechanical resistance and tenacity. The starch film with 30% Gly content and PAH 1% exhibited an elastic modulus and tensile strength of the same order of magnitude of LPDE and a good elongation at break even if lower than LDPE. The introduction of the *T. obliquus* biomass as bioactive filler endowed the films with a remarkable antioxidant activity, which increased proportionally to the microalgae content (up to 1%), maintaining at the same time good physico-mechanical properties of the film. *T. obliquus* biomass is a source of several bioactive compounds, easily producible at large industrial scale, without the needing for arable lands. Specific biodegradability tests were not carried out in this study. However, we expect a biodegradability behavior similar to the native starch, or even higher when the microalga is introduced in the film due to the presence of nitrogen, which can act as a nutrient for microorganisms. This study has shown how its antioxidant potential can be exploited in the production of a bioactive starch-based films. The obtained films hold the potential to be used as flexible packaging.

## Figures and Tables

**Table 1:** Degradation temperature (T_d_), soluble fraction, equilibrium water swelling, contact angle and water vapour transmission rate (WVTR) of pure starch, S_GlyX and S_PAH1_GlyX films.

Sample	T_d_ (°C)	Soluble Fraction (%)	Equilibrium Water Swelling (%)	Contact Angle (θ°)	WVTR (g/h m^2^ mm)
Starch	300	3 ± 1	110 ± 10	85 ± 1	3.2 ± 0.2
S_Gly10	300	10 ± 2	135 ± 15	70 ± 1	2.0 ± 0.2
S_Gly20	300	15 ± 2	120 ± 10	63 ± 2	1.2 ± 0.2
S_Gly30	300	35 ± 3	125 ± 8	61 ± 3	0.6 ± 0.1
S_PAH1	275	1 ± 1	150 ± 10	77 ± 1	2.2 ± 0.2
S_PAH1_Gly10	270	21 ± 2	130 ± 10	79 ± 5	2.4 ± 0.2
S_PAH1_Gly20	273	20 ± 3	125 ± 7	75 ± 3	3.1 ± 0.1
S_PAH1_Gly30	290	22 ± 2	90 ± 10	84 ± 3	2.6 ± 0.1
Gly	250	-	-	-	-
PAH	350	-	-	-	-

**Table 2:** Elastic modulus (E), Tensile Strength (TS), Elongation at break (EB) and Tenacity (T) of S_GlyX and S_PAH1_GlyX films.

Sample	E (MPa)	Tensile Strength (MPa)	Elongation at Break (%)	Tenacity (MPa)
S_Gly10	310 ± 100	30 ± 2	3.2 ± 0.8	0.34 ± 0.05
S_Gly20	400 ± 200	34 ± 8	4 ± 1	1.1 ± 0.3
S_Gly30	25 ± 1	1.9 ± 0.4	13 ± 2	0.11 ± 0.01
S_PAH1	1100 ± 200	35 ± 5	4 ± 1	0.97 ± 0.08
S_PAH1_Gly10	300 ± 100	6 ± 1	35 ± 2	1.5 ± 0.5
S_PAH1_Gly20	30 ± 10	3.5 ± 0.5	45 ± 3	2.2 ± 0.1
S_PAH1_Gly30	60 ± 20	4.0 ± 0.8	78 ± 2	2.6 ± 0.2

## Data Availability

Not available.

## Abbreviations

S = starch
Gly = Glycerol
PAH = poly(allylamine) hydrochloride
S_Gly10 = Starch film with 10% Glycerol
S_Gly20 = Starch film with 20% Glycerol
S_Gly30 = Starch film with 30% Glycerol
S_PAH1 = Starch film with 1% PAH
S_PAH1_Gly30 = Starch film with 1% PAH and 30% Glycerol
S_PAH1_Gly30_0.1 = Starch film with 1% PAH and 30% Glycerol and 0.1% microalga
S_PAH1_Gly30_0.5 = Starch film with 1% PAH, 30% Glycerol and 0.5% microalga
S_PAH1_Gly30_1 = Starch film with 1% PAH, 30% Glycerol and 1% microalga
